# The three-step persuasion model on YouTube: A grounded theory study on persuasion in the protein supplements industry

**DOI:** 10.3389/frai.2022.838377

**Published:** 2022-10-12

**Authors:** Jayanshi Tripathi, Roelof A. J. de Vries, Mailin Lemke

**Affiliations:** ^1^Human Media Interaction, University of Twente, Enschede, Netherlands; ^2^Biomedical Signals and Systems, University of Twente, Enschede, Netherlands; ^3^Department of Human Centered Design, Delft University of Technology, Delft, Netherlands

**Keywords:** persuasion, behavior change, grounded theory, qualitative research, interpretivism, YouTube influencers, persuasive techniques, social media

## Abstract

Persuasion can be defined as an active attempt by a person to change the behavior and attitudes of others. The purposive attempt to influence one's behavior can originate from different areas, and people who are able to do so are often referred to as influencers. Social media platforms such as Instagram or YouTube have become crucial platforms for influencers who generate their income by recommending products and services to their followers, including cosmetics, multimedia articles or clothing. Studies indicate that influencers actively try to persuade the viewer to adopt specific desirable behavior by strategically altering their displayed behavior on social media. Such strategies have mainly been explored in the context of beauty products, where lack of expertise and misinformation might have few negative consequences. Less is known about strategies used in a health-sensitive context, such as nutritional supplements. This research addresses this gap and aims to understand persuasive techniques used by health professionals on YouTube to promote the use of protein supplements. This study is based on an interpretive paradigm using interpretive grounded theory to analyze 60 YouTube videos. We developed a three-step model of persuasion for YouTube videos consisting of the steps: reaching the message, staying on the message, and performing the action that the persuader desires. Our analysis resulted in five core themes that contributed to the persuasiveness of the analyzed YouTube videos. These themes included: Quality, curiosity, engagement, concretization, and genuineness. We conclude the paper with reflections on our model's theoretical and practical implications.

## 1. Introduction

Many theories try to explain why humans behave the way they do and how their behavior can be steered toward an “ideal” or desired one. The widely cited Social Learning Theory is a comprehensive model that integrates behavioral and cognitive learning theories. The theory proposes that people can learn new behaviors through observation and imitation of others instead of learning by direct experience (Bandura and Walters, 1977). This modeling of the ideal behavior can originate from different stimuli including people who demonstrate the desired behavior; verbal instructions that outline the behavior, or symbolic means where the desired behavior is embedded in the social context, including communication technology such as television (Bandura and Walters, 1977). In the technology-driven lives of today, social media provides an opportunity to observe the behavior of others and use online information as a critical ingredient in making personal judgements, including purchase decisions (Alalwan et al., 2017; Jiménez-Castillo and Sánchez-Fernández, 2019).

If people adopt new behaviors by observing others, then those who are observed can promote an “ideal” behavior. In other words, by strategically altering their behavior, the person who is getting observed can indirectly persuade the observers to adopt specific desirable actions. This influence on people can be achieved differently, and so-called influencers who evoke a social influence on others originate from different social-cultural areas (e.g., sport, political or cultural sectors) (Khamis et al., 2017). The rise of social media has offered a new platform giving rise to a new kind of influencer, referred to as social media influencers (SMIs). SMIs commonly share insights into their private lives that create a sense of realness and intimacy which helps to monetise their personal image (Khamis et al., 2017). Their role can be described as a *hidden persuader* who commonly weaves commercial products into their private environment. Strategies include the development of carefully crafted personal branding, staged scenes in which either photos or videos are taken, and specific choreographies to influence the viewer's perception and behavior and encourage an imitation of the desired behavior (e.g., purchase of a particular product) (Ferchaud et al., 2018; Rohde and Mau, 2021).

The prominent role of social media in getting people to imitate others can be illustrated through several phenomena. For example, participating in the ice bucket challenge to raise awareness of the disease amyotrophic lateral sclerosis, taking part in the “trashtagchallenge” that encourages people to clean up polluted areas or the increased demand for avocado toasts. While the latter example might appear trivial, does the avocado toast illustrate how a product can become popular on social media platforms. When health professionals began publicizing the idea that the mono-saturated fats in avocados may have health benefits, celebrities and Instagram influencers started extensively glorifying avocado toast as a healthy, simple breakfast (Gagnon, 2021). In 2019, the avocado toast had at least 1.2 million Instagram hashtags, and the prices soared rapidly in restaurants and cafes, as did people's willingness to pay unreasonable prices for it (Orenstein, 2016).

Social media can extensively facilitate the process of observation and imitation of others, as illustrated with the avocado toast example. The topic that SMIs focus on varies, and even everyday products such as food items, can be influenced by SMIs (Al-Harbi and Badawi, 2021). Still, the topic of food choices is also an area where scientific advice can collide with the one provided by SMIs and, in a worst-case scenario, affect one's health (Khamis et al., 2017). This research aimed to explore the persuasive techniques used by SMIs, in particular YouTube content creators who promote the use of protein supplements. Protein supplements were initially predominantly used by bodybuilders but are now used by many people as a meal replacement due to associated health benefits and for weight reduction (Bartels and Miller, 2003; Samal and Samal, 2018). The main reason protein supplements were chosen as the context for this study was the rapid growth in the global consumption of protein supplements in the last few years and its continued projected expansion until 2028 (ResearchandMarkets, 2021; MordorIntelligence, 2022). Furthermore, by popularizing the health and wellness movement, social media platforms have played a pivotal role in the growth of the protein supplements market (Mahrra, 2015; Heitner, 2016; Hilkens et al., 2021).

For this study, we chose to focus on long-form videos on the social media platform YouTube, which is the second most popular website on the internet (Similarweb, 2022). Our selection was based on reports indicating that nearly two out of three consumers use online videos to get purchase ideas and inspiration (Google, 2018). Six out of 10 YouTube subscribers report that they would follow the purchase recommendation of their favorite creator over that of famous TV and movie personalities (O'Neil-Hart and Blumenstein, 2016). We concentrated on long-form videos produced by SMIs as video blogs (short vlogs). As part of vlogs, influencers commonly talk to the camera about a personal topic while sharing with the viewer how they live and work (Rohde and Mau, 2021). The recorded video is then edited and uploaded to the internet, where viewers can rate and/or comment on it (Frobenius, 2011).

The current study makes several contributions to the ongoing debate regarding the role of SMI and concrete persuasive strategies used in this context of food with an associated health benefit. We argue that the model we developed can be used as a source of guidance and inspiration to researchers in human-computer interaction (HCI) and marketing who actively create new persuasive technology. For example, a detailed understanding of techniques for online persuasion could guide social media content creation for positive food consumption. Furthermore, researchers and practitioners could use the model to design persuasive interfaces, apps or even chatbots.

The manuscript is structured as follows: We first outline current findings regarding SMIs and persuasive strategies. We then describe the methodology and steps undertaken during the iterative data collection and analysis process. In a consequential step, we describe our three-step persuasion model. We conclude the paper with a discussion of the model and point out potential limitations.

## 2. Background and related work

In this section, we describe related work conducted in the context of SMIs. We would like to emphasize at this stage that we followed a grounded theory approach that focuses on the literature review once the data collection and analysis are completed to avoid bias. Therefore, the outlined frameworks and theoretical findings were not taken into consideration as part of the study design and analysis process but are presented to provide an overview of current research efforts.

YouTube can evoke a sense of community among followers (Rotman et al., 2009), especially among people who subscribe to a particular channel (Cocker and Cronin, 2017). Interactive features of the platform, such as the rating function of videos, influence consumer perception toward the advertised products (Hu and Yao, 2021). Furthermore, the comment function that YouTube provides facilitates social interaction and lets the video creator and viewer interact and share ideas (Xiao et al., 2018). From a marketing point of view, these social media platforms and SMIs can be used as a persuasive tool to facilitate consumer engagement, provide information and influence consumer decisions and behavior (Hemsley and Mason, 2013; Jiménez-Castillo and Sánchez-Fernández, 2019; Ki and Kim, 2019; Hu and Yao, 2021; Sokolova and Perez, 2021).

A number of empirical studies have explored how persuasive strategies used by SMIs influence consumer perception (Vrontis et al., 2021). For example, Sokolova and Kefi (2020) explored how physical attractiveness, attitude homophily and social attractiveness influence credibility and parasocial interaction, which in turn can influence the viewer's purchase intention. Their findings on beauty and fashion SMIs suggest that credibility and parasocial-interaction with the SMIs influences the purchase intention. Other studies have explored the multi-modal experience of vlogs, including verbal, visual, acoustic and para-verbal descriptors to predict the persuasiveness of online social media content (Park et al., 2016) or investigated the linguistic style and level of excitement and passion expressed by the SIMs (Lee and Theokary, 2021). Other findings suggest that the social and physical attractiveness of the SMI seems to play a role in the context of evaluating the endorsed product as well as the viewer's motivation to watch the content, e.g. vlog (Liu et al., 2019; Torres et al., 2019). These findings indicate that SMI-based persuasion includes elements viewers might be quickly aware of (e.g., what is said) as well as covered elements (e.g., tone of the presentation). One way to explain such findings is by relating them to the dual-process persuasion models, which link persuasion to social cognition. The models outline that information is processed in two distinct ways. On the one hand, information is processed through central processing which is a more effortful but systematic approach. On the other hand, information is processed through peripheral and heuristic cues (e.g., attractiveness) which can in turn evoke a persuasive effect. These cues usually are less resource demanding and less analytical (Xu, 2017).

The perception of a presented product as part of a vlog is likely influenced by the setting of the video, style of communication and detail and amount of information presented. For example, Rohde and Mau (2021) used the social influence heuristics proposed by Cialdini (2008, 2016) to analyze long-form videos on YouTube produced by SMIs to market their brands and products. They illustrate how the heuristics of reciprocity, social proof, consistency, scarcity, liking, authority and unity are incorporated into several creative techniques. For example, in the context of liking, positive emotions such as humor or surprise are used by SMIs, as well as elements that evoke an impression of familiarity and similarity, such as using the same setting for a video (Rohde and Mau, 2021). Xiao et al. (2018) developed a heuristic-systematic model outlining that the factors of trustworthiness, social influence, argument quality, and information involvement influence the aspect of information credibility, which in turn influences video and brand attitude. Ladhari et al. (2020) explored how homophily (i.e., attitude, value, background, and appearance), the vlogger's expertise, and emotional attachment to the vlogger influence perceived popularity and consequent purchase decision. Their findings in the context of beauty advice suggest that viewers who perceive a vlogger to share similar attitudes (e.g., thinking and behavior) and similar values are likely to feel a stronger emotional attachment. However, it also seems that viewers do not need to feel that they share the same appearance or social background as the vlogger to establish an emotional bond. In the context of beauty advice, it also appears that the factors of technical expertise (e.g., qualification) do not influence the vlogger's popularity (Ladhari et al., 2020; Wiedmann and von Mettenheim, 2020).

However, while a lack of expertise might have little influence in the context of beauty products, it might play a role in health-related products. For example, the American cardiothoracic surgeon “Dr Oz” who is known as a television personality with millions of viewers, significantly influences his viewers' perception of the advertised health products and treatments (Bootsman et al., 2014; Stecula et al., 2022). The advice provided as part of the show commonly focuses on nutritional advice and has been criticized for its lack of evidence supporting the claims. It seems that just a third of the recommendations are based on believable to somewhat believable evidence (Korownyk et al., 2014). This example shows that people's trust in medical authorities also extends into the digital context, and medical professionals can take on the role of influencers. In this context, it has been noted that the topic of SMIs and healthy eating/use of supplements is an under-explored research area (Folkvord et al., 2020; Hilkens et al., 2021; Von Mettenheim and Wiedmann, 2021). Therefore, it remains unclear what kind of persuasive strategies are used in this context to persuade the viewer to purchase products that offer nutritional benefits and promise improved health (e.g., weight loss). In addition, a recent systematic literature review of SMI marketing strategies has pointed out that the majority of studies conducted in this area focus on quantitative studies with a strong focus on theory testing rather than theory development using qualitative methods (Vrontis et al., 2021). With this study, we aim to address this gap and focus on a qualitative approach to explore the different strategies used by SMIs on YouTube to promote nutritional supplements.

## 3. Methods

For this qualitative research, we chose an interpretive research paradigm (Johannesson and Perjons, 2014). The central idea in interpretivism is that there is no single reality, and instead, realities are created differently by individuals based on their subjective experiences (Johannesson and Perjons, 2014). The interpretive paradigm emphasizes social context and social constructions such as language as critical elements in understanding human behavior (Pham, 2018). Qualitative research is typically associated with this paradigm (Charmaz, 2003). We chose grounded theory as a research methodology because it is aligns with the principles of interpretivism (Cassiani et al., 1996).

Grounded theory focuses on theory generation based on systematic data collection and analysis process (Noble and Mitchell, 2016). The methodology is suitable when the research aim is to produce or construct an explanatory theory behind a social process (Charmaz, 2003). Grounded theory offers structured, sequential guidelines for performing the data collection and analysis process. Its inductive nature allows subjective interpretations during data analysis, which aligns with the epistemology of interpretivism chosen for this study. For example, grounded theory enables the researcher to go beyond the main collected data and form interpretations of the context from which the data is collected.

Grounded theory comes in three flavors: classic, interpretive, and constructivist (Sebastian, 2019). We chose interpretive grounded theory that advocates the emergence of the research question from the research process itself rather than starting with a tightly defined hypothesis (Charmaz, 1990; Birks and Mills, 2015; Sebastian, 2019). This approach differs from traditional research approaches that commonly include formulating a research question or hypothesis at the start of the study. As part of interpretive grounded theory, the researchers begin with a rather broad research question to define the research context and guide the initial stages of the research. This general question is narrowed down and finalized later in the research process. We started with the following (broad) research question:

***Broad RQ:*** *What persuasive strategies are used on digital platforms to encourage the usage of protein supplements?*

In one aspect of the methodology, we focused on the classic grounded theory stance: This aspect concerns the literature review. Unlike most research methodologies where a literature review is used to frame the research question or hypotheses (Creswell and Creswell, 2017), Glaser and Strauss (Foley et al., 2021) argue strongly against early exposure to existing theories. They argue that a literature review could create preconceptions, hindering the natural emergence of categories from the data. Glaser (1998) also points out that given the unpredictable nature of grounded theory, the literature that is most relevant to the research cannot be determined at the beginning. It is, thus, inefficient to delve into an extensive review. On the other hand, the researcher likely experiences some kind of bias. For example, researchers might have some preconceived notions about the topic or theoretical awareness (Charmaz, 1990; Cutcliffe, 2000; McGhee et al., 2007). We, therefore, carried out a literature review after the intermediate analysis phase to reduce bias during the initial analysis process. The literature review helped to compare the results of this study with existing theories of persuasion. We extensively exercised reflexivity during this phase through memo writing, noting down the ideas we were exposed to through the literature, and reflecting on how they may influence the research results. Reflexivity helped in the process of reducing potential theoretical bias.

The rest of this section will introduce essential grounded theory concepts and outline how we used them in this study. Since grounded theory is an iterative methodology, there were multiple data collection, coding, and abstraction cycles before we refined our theory.

### 3.1. Data collection

We chose audio-visual data uploaded to YouTube as a data source. The data included the videos themselves, specific characteristics of a video (e.g., thumbnails and title) and its creators, such as age, gender, and professional occupation. This kind of additional information helps form a thorough understanding of the social context in which the video is embedded. Furthermore, the comment section also provided contextual insight into the video's audience.

Grounded theory uses different kinds of sampling techniques, often starting with purposive sampling (Lavrakas, 2008; Moser and Korstjens, 2017) and later on using theoretical sampling to fill in and extend the initial theoretical constructs (Charmaz, 1990; Moser and Korstjens, 2017). The analysis and data collection process is, therefore, done in parallel with theoretical sampling. As opposed to choosing a sample for population representation, theoretical sampling refers to using the results of the existing data analysis to determine what type of data to collect further and from where (McCann and Clark, 2003). This tightly-knitted, iterative data collection and analysis process plays two salient roles in the research process. First, it gives rise to a well-defined research question. Second, the iterative, parallel data collection and analysis process determines when the data's theoretical saturation has been achieved. Theoretical saturation refers to the situation when the analysis of new data does not yield new categories (Noble and Mitchell, 2016). With the achievement of theoretical saturation, data collection stops. Once we had reached theoretical saturation, our focus shifted to refining the collected data to produce a substantive theory.

### 3.2. Data analysis

We used two different instruments as part of the grounded theory development. The first one, labeled the “Audio-Visual data as an Object of analysis” (Figueroa, 2008) helped in the analysis of the audio-visual material. The second one, titled “Ünlü-Qureshi Analysis Instrument” (Qureshi and Ünlü, 2020) was used in the abstraction and theory development process.

#### 3.2.1. AVO perspective

In grounded theory, researchers record social phenomena and processes by recording videos. The video, from this perspective, is seen as a medium rather than a specific final product and phenomenon in its own right (Figueroa, 2008). However, in this study, we regard YouTube videos as the product of persuasive attempts by the SMI. Therefore, the videos consisting of audio-visual data were used as the primary data source. This perspective of viewing audio-visual data as the product of the phenomenon is what Figueroa (2008) labels as the “audio-visual data as an object of analysis,” in short, AVO-perspective. This perspective emphasizes that the audio-visual material should be attended to on two levels. The first level focuses on the actors and their displayed interactions, and the second level should consider the producers of the video and the strategies they used. The AVO-perspective recommends viewing the video first at least once entirely to obtain a general impression. This initial understanding of the material should then be used as the lens through which individual fragments of the audio-visual text can be analyzed and coded. Thus, the analysis takes place for each video on two levels: a contextual and a specific level.

**Figure 1:**
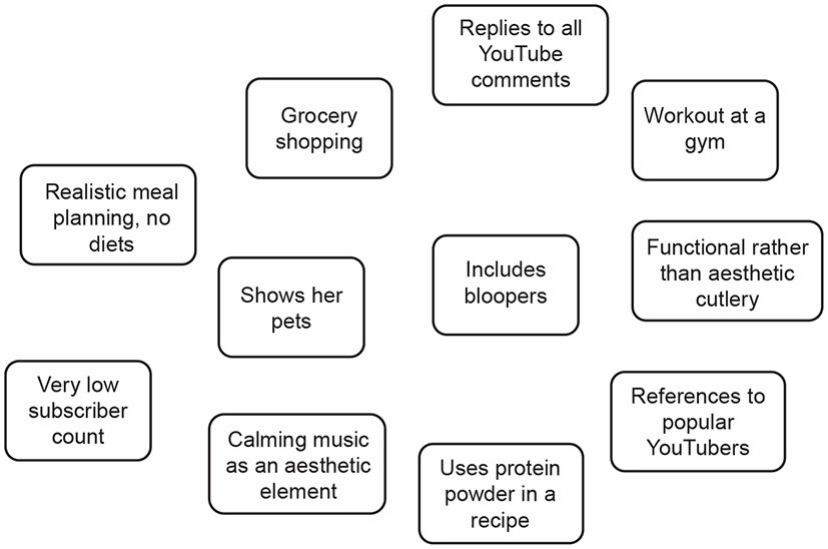
A sample situational map constructed during level 1 of the AVO analysis of a 7-min YouTube video.

The first *contextual level* of analysis focused on establishing a general impression of the video. This was done by viewing the video as well as by reviewing additional information, including thumbnails, video titles, demographics of the targeted audience, disclosed financial motives of the content creators, subscriber count of the channel, the environment in which the video is shot, aesthetics, and the dressing style of the people in the video. We used situational maps (Clarke, 2003), instead of the impressionistic notes suggested by Figueroa (2008) to systematize and document the first level of analysis. Our situational maps were diagrams that contained all contextual information observed during the first watch of the video in a condensed and visual format (see Figure 1). The graphic nature of situational maps allowed a concise notation of the observed factors and put them in a relationship.

The second *specific level* of analysis in the AVO-perspective focused on coding elements of the video that we perceived as relevant after the first level. Coding was conducted in a line-by-line fashion on the subtitles of the YouTube videos. We also considered the visuals and verbal factors such as tone and pitch of the voice while coding. For example, when a protein powder was shown on the kitchen counter during a video snippet that was not related to protein or nutrition, one of the codes assigned to the text of that snippet was “product placement.” On the one hand, provided this detailed observation many new insights into the data, thereby enhancing the richness and depth of the analysis. On the other hand, led these details to an extensive amount of information that needed to be organized and systematically incorporated during the coding process. To facilitate this process, we used memos. Writing memos is of chief importance in grounded theory research, as they help researchers understand the data by encouraging them to reflect on it and document their thoughts while coding. They also have a significant role in tracking the development of theory (Triad3, 2016), as we will outline in the following section.

#### 3.2.2. Ünlü-Qureshi analysis instrument

The way codes are developed, and a theory is formulated can differ between the different schools of grounded theory (Sebastian, 2019). The coding approach used in this research was based on the “Ünlü-Qureshi Analysis Instrument,” which entails four steps of data analysis. The instrument allows being used by different grounded theory approaches by providing an analytical and flexible process (Qureshi and Ünlü, 2020). The coding instrument encompasses four essential items: (1) code; (2) concept; (3) category; and (4) theme. A *code* indicates what a data extract represents or is. Codes are assigned directly to pieces of the data. Each *concept* represents an idea shared by multiple codes. Concepts are grouped into a *category* when they share a common property or characteristic. Finally, a *theme* is the most abstract item in coding, and it captures an overarching idea shared by all the codes, concepts, and categories. The instrument includes three different coding stages: (1) Open coding stage, including initial data; (2) selective coding stage, including focused data; and (3) theoretical coding.

**Open coding stage:** During this phase, the data is analyzed in an open way to develop codes, concepts, categories and theme(s). The researcher starts the process by familiarizing themselves with the data and coding the data excerpts by adding short labels to the data (Similar to “Open-coding” in interpretive grounded theory Sebastian, 2019) until saturation is reached. In a consequent step, the codes are compared and contrasted and then clustered under a concept. This step is repeated for concepts to achieve a higher level of abstraction leading to the development of categories. At the end of this process, all components should hint toward one umbrella term, referred to as a theme.

**Selective coding stage:** In this phase, the focus is on strengthening and refining the codes, concepts and categories discovered in the initial analysis (This is similar to “Axial-coding” Walker and Myrick, 2006). Extensive data collection and analysis are not the main focus of this phase. However, some additional data can be analyzed during this stage, and new concepts and categories may emerge. The process is completed when saturation is reached.

**Theoretical coding stage:** The goal of this coding stage is to examine, refine and strengthen the relationships between the themes and their components (Similar to “Selective-coding” in interpretive grounded theory Walker and Myrick, 2006). As part of this stage, possible links between themes are examined as part of the “theoretical sorting.” A second task includes the formulation of hypotheses by describing the theory and its components.

We used constant comparative analysis as part of the process, which is an integral component of grounded theory (Kolb, 2012). Constant comparative analysis (CCA) is the process of comparing and contrasting various codes to form categories. CCA is combined with memo-writing to document the understanding of the theoretical constructs obtained through constant comparison. CCA is used throughout the data analysis process to continually examine relationships and abstract the data to develop a theory.

### 3.3. Procedure

The first author conducted the data collection and analysis. Initial codes and themes were discussed among the three authors and refined.

#### 3.3.1. Open coding stage

The first author started the data collection and analysis process in May 2021 by searching for “*What I Eat in a Day”* videos through the YouTube search box. The term was chosen for the purposive sampling based on news reports indicating their popularity in the realm of fitness content on social media (Emanuelli, 2020; Johnston, 2020). The results of the query were then sorted into two metrics. We used view count and rating, which are two correlated popularity metrics on YouTube (Chatzopoulou et al., 2010). The top five videos were chosen from these two metrics, leading to 10 videos for the initial data analysis. The first author analyzed the 10 videos.

We noticed two problems during the contextual focus of the AVO-perspective of the sampled data. First, the five videos chosen based on the rating metric had very few views, in the range of 200–1,000. This observation contradicts statements that the popularity metric of rating and view counts on YouTube are correlated (Chatzopoulou et al., 2010). The subscriber counts of these videos were also between one to five thousand. Because there were many nutrition channels with more than 50,000 subscribers and 100,000 views, we decided that the five top-rated videos were not representative of most nutrition channels in terms of popularity. Therefore, we chose not to proceed with level two of the AVO analysis which includes the specific coding of these five videos.

The second problem was that three of the other five videos chosen based on view counts were centered around celebrities and seemed to be made for entertainment rather than for offering serious nutritional advice. The two remaining videos were about professional bodybuilding and sumo wrestling champions. An average person's diet and dietary needs are likely not comparable to such professionals. We did not proceed with coding the videos due to doubts about representation.

This situation led us to the following question “From which kind of videos do people take nutritional advice?”. This question is also crucial because following wrong health-related information may have serious repercussions. So, to answer the above question, we conducted exploratory, informal, open-ended conversations with the target audience as a means of theoretical sampling (Glaser et al., 1968) to refine our search terms. The conversations were not recorded nor used as part of the data analysis process but rather helped refine the search terms. The interviewees were university students (ages 19–24 years old) with (self-reported) moderate to high awareness of their dietary requirements. They were primarily asked about how they assimilate nutritional information on the internet. In addition, we explored their general attitude toward nutrition professionals online. This exploratory step indicates that health is an essential aspect of people's life quality and that there is an appreciation of content creators' professional expertise. Credibility was also associated with titles such as a nutritionist and (registered) dietitian. We, therefore, refined our research question to:

***RQ:*** *In YouTube videos made by health professionals, which factors contribute to persuasion toward protein supplements?*

During the conversations, it was also mentioned that *reaction videos* were particularly informative. In these videos, a health professional reacts to the *What I Eat in Day* video of popular figures such as influencers and celebrities. The health professional evaluates the nutritional quality of the recommended diets and gives professional advice on how to improve them. We began collecting and analyzing reaction videos based on this suggestion.

The videos were chosen by searching for “nutritionist reacts” and “dietitian reacts.” The term “protein” was added to these queries as well. Then the results were sorted based on view counts which is YouTube's fundamental parameter indicating popularity (Chatzopoulou et al., 2010). We selected 10 videos based on this process (with and without the term “protein”). We reviewed the videos and excluded instances that had no relevance to protein supplements (e.g., no discussion of protein).

**Figure 2:**
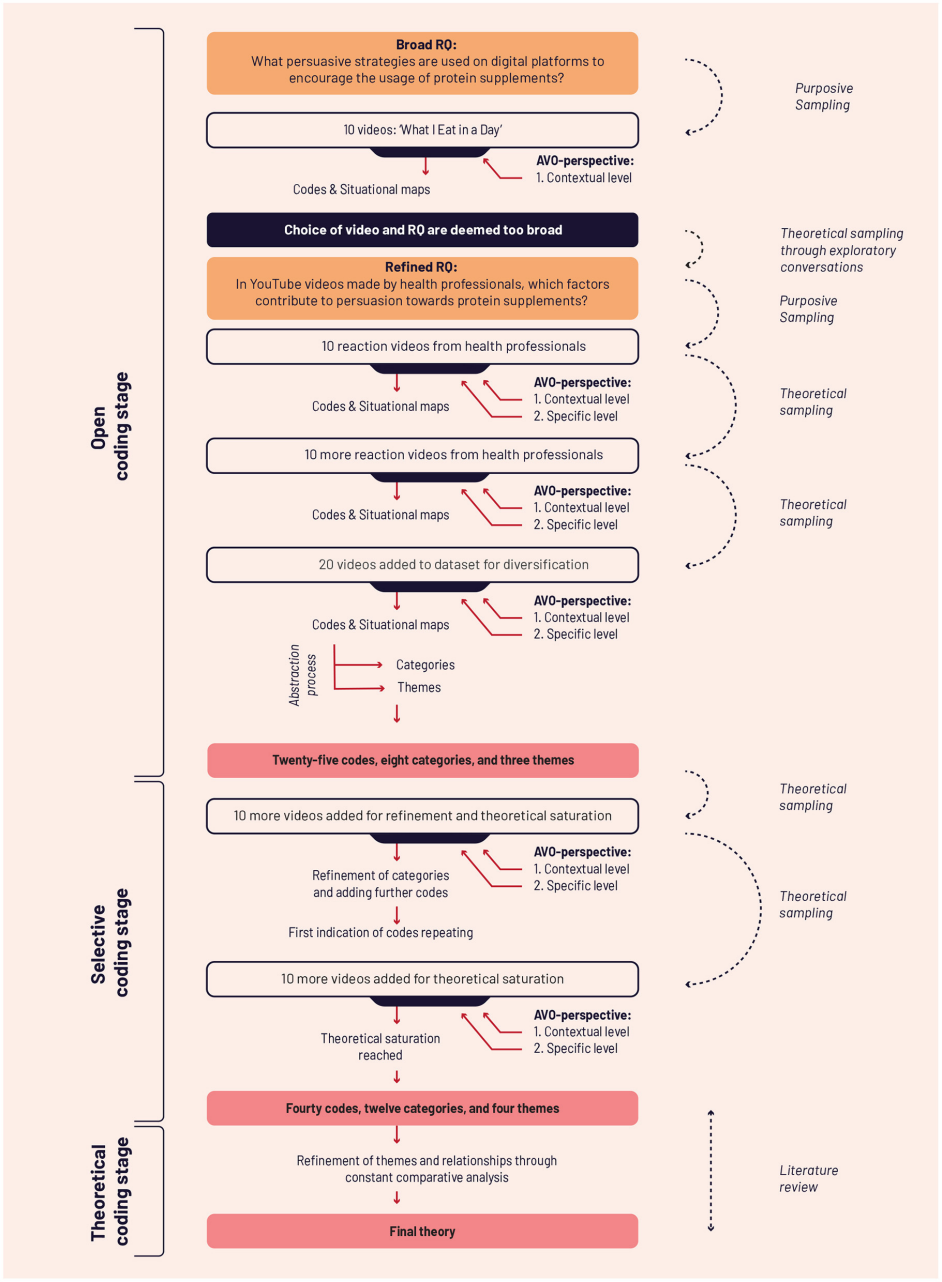
The complete data collection and analysis process of this study.

After we had coded a total of 20 reaction videos (split up into analyzing 10 videos per step, see Figure 2), we observed that the analyzed videos lacked diversity in terms of the targeted audience. For example, most videos were made by female creators, from only a handful of YouTube channels and followed a similar content format. To increase diversity and enhance the representative nature of our included data, we used theoretical sampling following two steps. First, we modified our queries to only the words “nutritionist protein” and “dietician protein.” This revealed various new types of videos. For example, there were videos where health professionals reviewed protein bars. There were also videos made by the official channels of popular supplement fitness lines which featured health professionals. The second step of the theoretical sampling was searching for Google's most popular fitness channels. Through this step, we discovered fitness channels with large followings that health professionals did not create, but that *featured* numerous health professionals. The creators of these channels were bodybuilding champions and well-known trainers. We analyzed 10 videos from such channels. The criterion for the video selection was the number of subscribers to the channel. The themes across the 10 chosen videos varied. We chose videos that had an increased involvement of health professionals and featured protein supplements to make sure that the data was relevant to our research question.

We analyzed 20 additional videos following this process, resulting in 40 videos analyzed during the open coding stage (not counting the 10 videos during purposive sampling). The first author reviewed the developed codes to search for concepts and categories by increasing the level of abstraction. We noticed in the process that using three levels of abstractions (code, category and theme) was more effective and sufficient in the context of this study. We, therefore, did not develop concepts during the analysis process.

The abstraction process concluded with 25 codes and eight categories. As the last step of the open coding, the researcher should ideally select a single core theme. However, despite repeated abstraction, we did not find one core theme that adequately explained most of the data. Therefore, we ended the initial analysis by selecting three themes.

#### 3.3.2. Selective coding stage

During the second stage of the coding process, the goal was to add more data to achieve theoretical saturation and refine the categories and themes. We, therefore, added 10 more videos to our analysis. We selected these videos based on the criteria described above. After these 10 videos, we noticed codes starting to repeat. Furthermore, the number of categories increased from eight to twelve, codes increased to thirty, and a new theme emerged. We analyzed 10 additional videos, after which we were confident of having reached theoretical saturation of the data, completing our selective coding stage.

#### 3.3.3. Theoretical coding stage

Finally, in the theoretical coding stage, we focused on examining the relationships between the four core themes and the 12 categories. Through constant comparative analysis, we established multiple links between the categories. We also reviewed the literature as part of this process to refine the conceptual elements of our model. The result of this stage was a three-step model of persuasion, which we present in the next section.

## 4. Results

### 4.1. The three-step model of persuasion

**Figure 3:**
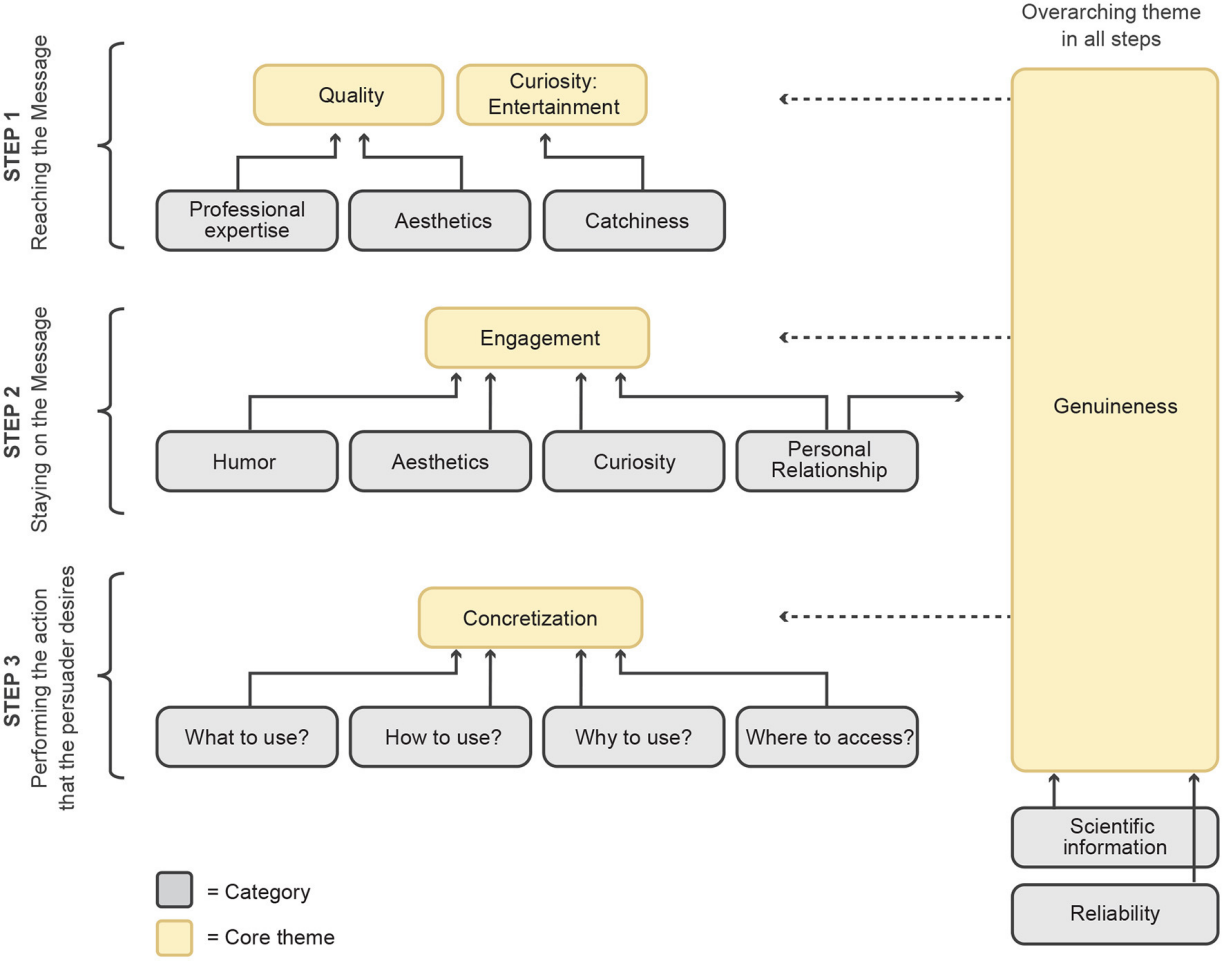
The three-step model of persuasion.

Our model proposes that on YouTube, persuasion is a process that can be divided into three main steps: (1) “Reaching the message”; (2) “Staying on the message”; and (3) “Performing the action that the persuader desires,” (see Figure 3).

#### 4.1.1. Step 1: Reaching the message

The first step in the model focuses on the need for the video to be noticed by the target audience, therefore, *reaching the message*. This step is crucial because there is an abundance of videos on YouTube related to fitness and nutrition. For the viewer to see the persuasive message embedded in a given video, they should select it first amongst millions of other videos. Through our literature review conducted during the theoretical sampling stage, we noted that thumbnails, titles, and categories are influential criteria in the decision making process (Foster, 2020). The situational maps constructed during data analysis revealed three common features: (1) *Professional expertise*; (2) *Aesthetics*; and (3) *Catchiness*. These three categories were grouped under the two themes “Quality” and “Curiosity: Entertainment” (Figure 3).

We see professional expertise and associated credibility to be linked to the characteristic of the video source, including credentials and years of professional experience of the video creator. Such persuasive features were, for example, included in the video description, including titles such as “Nutritionist reacts to What I Eat In A Day,” “What a Registered Dietitian Eats in a Day” or “Best Protein Bars according to a Dietician.”

The second persuasive element—aesthetic appeal—is arguably subjective. We observed during the analysis process that most thumbnails of the videos consisted of high-quality pictures, including some aesthetic features. For example, by including colorful food images or showing the video creator, often by displaying toned muscle areas such as abs or arms as part of the thumbnail.

The third and final persuasive feature focuses on catchiness to provoke the curiosity of the viewers, which in turn contributes to aspects of entertainment. In this context, it is essential to remember that YouTube is a social media platform, and people use it predominantly for entertainment. Factors such as professional credibility can enhance the viewer's trust in the video and perceived level of quality, but entertainment can contribute to the viewer's desire to watch the video. Our analysis of thumbnails and titles revealed two major ways entertainment is incorporated into the videos. The first way is by triggering curiosity through the tiles, and the second way is by referencing celebrities. Good examples are the video titles “Why Gwyneth Paltrow's “INTUITIVE FASTING” is DANGEROUS (And I Have to Call Her Out…!)” and "Dietitian Reacts to Ashley Graham What I Eat In A Day (Wow, You'll be Surprised, Too…)”.

To conclude, the first step of our model focuses on encouraging viewers to click on the video and “reach the message.” This step consists of establishing the perception of quality by linking it to professional expertise and aesthetic features. This step also aims to persuade viewers by evoking elements of curiosity and entertainment.

#### 4.1.2. Step 2: Staying on the message

Once the viewer has decided to start watching the video, it is important that they *stay on the message*. We identified “Engagement” as the main theme of this step. We define engagement to take place when the viewer of a video experiences any emotion while watching it. In this research, we focus on positive emotions such as enjoyment. We identified the four categories of (1) *Humor*; (2) *Aesthetics*; (3) *Curiosity*; and (4) *Personal Relationship* (see Figure 3) to be used as a kind of encouragement to continue watching the video until the persuasive message (e.g., product placement) has been presented by the video host. For example, for the category humor, we noticed that many content creators have "inside jokes" with their viewers to create engagement. Self-deprecating jokes were also use frequently by the YouTubers. Another strategy for creating engagement was when the audience were explicitly asked to comment on the video through prompts such as “comment below if you like your bread lightly toasted or toasted multiple times” and “let me know if you are a coffee person or a matcha person.”

#### 4.1.3. Step 3: Performing the action that the persuader desires

Once a viewer has decided to watch a video and paid close attention to its content, they need to do the desired action, such as buying the presented protein supplement. Therefore, the third step of our model focuses on performing the action that the persuader desires. Our analysis suggests that persuasive features concentrate on describing the desired actions precisely and in a concrete manner. The main theme of this step, labeled “Concretization” comprised of four categories: (1) *What to use*; (2) *How to use it*; (3) *Why to use it*; and (4) *Where to access it*.

Concretizing an action entails giving compelling arguments on “Why” the viewer should do the activity and what its results could look like. This display can be seen as a source of motivation for doing the desired action. In YouTube videos, clips of men and women with exercised muscles lifting heavyweights in a gym indicate the anticipated results of consuming protein, including increased muscle strength and a “pleasant” physical appearance. Mentioning academic articles as part of the video can be seen as a persuasive strategy that convinces the viewer to buy protein supplements because they help establish the product and its associated benefits to be trustworthy and backed by scientific studies. The theme of concretization also includes telling the audience precisely “What” to purchase. For example, a video might convince a viewer to buy a supplement because of the category “Why” based on the persuasive features used by the video creator. However, this particular viewer could still get confused and overwhelmed by the vast choice of protein supplements, thus giving up on buying them.

The category of “Where to access” focuses on persuading the viewer to buy a specific brand of supplements and reducing the risk of feeling overwhelmed during the purchase decision process. Discount offers and links to e-commerce websites for protein supplements can encourage and simplify the process of executing the purchase.

The fourth theme also describes “How to use” the product. As part of the video, the viewer should receive information that gives a clear idea of how they could practically and realistically incorporate this product into their everyday life. This included providing quick recipes and concrete meal plans that involved supplements.

#### 4.1.4. Overarching theme: Genuineness

Our model consists of one additional theme, labeled “Genuineness,” that does not fit exclusively into a single step but instead influences all three steps (especially step 2 and step 3). We define genuineness to be based on a natural, unstaged and trustworthy perception of the video. The persuasive message of the video should not come across as pushy, artificial or as a blatant advertisement. The theme of genuineness is influenced by the categories of *scientific information* and *reliability*. Furthermore, the category of personal relationships included in step 2 of our model contributes to elements of genuineness.

The persuaders (video creators) should appear to believe in what they are telling the audience. By appearing more relatable, shooting the video in a natural environment such as a kitchen and sharing personal stories, the video creators establish an image of being just an “average human being” that the viewer can relate to and less like a salesperson. By emphasizing one's flaws through strategies such as self-deprecating jokes, the video creator becomes more relatable to the viewer. Supplying the viewers with logical reasons (such as scientific information) for acting can contribute to genuineness. These factors help counteract any ulterior motives the video creator may have (e.g., being paid by the brand they advertise) and make the message more trustworthy. Genuineness can help the audience to perceive the video as trustworthy.

## 5. Discussion

When thinking of YouTube design, a term that comes to mind is “attention economy,” coined by psychologist and economist Herbert Simon (1996). The concept of attention economy refers to the fact that attention is a limited resource, and an abundance of information deteriorates attention. While social media platforms offer advantages to users and content creators, they also pose challenges. For example, a simple observation of the desktop interface version of YouTube reveals a large number of videos competing for the viewer's attention at any given moment. How the viewer is persuaded first to choose a particular video and then keep watching it often depends on several factors.

In this study, we used interpretive grounded theory to analyze the persuasive strategies employed by SMI as part of vlogs on YouTube to persuade viewers. We focused on protein supplements as a research focus due to their nature of combining food and health. Arguably, this context requires different persuasive strategies compared to SMI, where a level of expertise of the SMI is of little concern (e.g., beauty or fashion sector). In this context, it also should be noted that using social media to disseminate health-related messages is a topic where care should be exercised. For example, the online distribution of statements that are not backed up by scientific findings. In a worst-case scenario, this can affect an individual's health (Lau et al., 2012).

The model we developed consists of three steps that include four themes and 13 categories. We will first describe the theoretical contribution of the proposed model by comparing it to relevant related theories. In a consequential step, we will reflect on its practical application.

### 5.1. Theoretical implications

With this study, we contribute to the theoretical understanding of persuasive strategies used by SMI to promote protein supplements. Although we found overlaps between existing literature and various parts of our model, there was no single theory that took into account all the factors discovered in our research.

“Social learning theory” introduced by Bandura and Walters (1977) explains the underlying cognitive process during steps one and two of our model. The theory describes the process of human learning and factors that motivate individuals to acquire and retain a specific behavior. For example, the factor “Attention” included in social learning theory, explains how the viewer's attention is first captured because of the video creator's professional credibility and the thumbnail's aesthetic appeal during step one. The heightened attention will likely influence the viewer to watch the video during step two. The viewer pays attention to the video because of engagement or interest. Interest has been pointed out to increase attention (Burnham, 1908). The category of “Professional Expertise” that influences the theme of “Quality” during step one can also be linked to the principle of persuasion by Cialdini (2007) named “Authority.” The principle is based on the idea that people are likely to follow the lead of credible, knowledgeable experts.

Another factor of social learning theory referred to as “Motivation” can hint at why people keep watching the video during step two and could be prone to follow strategies provided during step three. First, motivation can be linked to our category of “Aesthetics.” For example, by showing people as part of the vlog who appear strong or attractive because of exercising and supplementing their diet with protein. Consequently, the viewers are motivated to meet the displayed body ideal. Motivation in this context can also be linked to the prospect of leading a better quality of life, as we noted during the analysis process that the SMI emphasized the health benefits of protein and its supplements multiple times in the videos. B.J. Fogg's model of behavior change also includes the factor of motivation to explain how behaviors can be evoked (Fogg, 2009). Suppose an individual shows low motivation to perform a specific behavior. In that case, the ability to do so (e.g., having the money to buy protein supplements) and the provided prompt (e.g., vlog by an SMI) will likely be insufficient to evoke the desired behavior (e.g., buying the protein supplement). Step three of our model consists of categories that function as prompts to elicit a specific behavior (Fogg, 2009).

In addition, the principle of persuasion named “Liking” can be seen to be connected to the theme of “Engagement” and our two categories of “Humor” and “Personal Relationship” (Cialdini, 2007). Lastly, shooting the vlog with a high level of aesthetics increases perceived enjoyment (Makkan et al., 2020), which in turn increases motivation (Hattingh et al., 2020).

The category of our model labeled “How to use” under the theme “Concretization” includes meal plans and recipe instructions for using protein supplements. This reduces the level of cooking skills and knowledge required to perform the behavior displayed by the SMI. In turn, the viewers feel encouraged to accomplish it. It is possible to explain this theme and category of our model with the factors of “Reproduction” and “Retention” of social learning theory (Bandura and Walters, 1977). “Reproduction” is often incorporated in the vlogs by simplifying the process of using protein supplements. At the same time, “Retention” is achieved by providing immediate means for executing the action by giving concrete instructions on how to access the product. This takes on the form of links to e-commerce websites in the description boxes of videos. Immediately following these links, the viewer can directly purchase the product.

Step three and the theme “Concretization” can also be linked to the “Construal Level Theory of Psychological Distance,” which says that an object is thought of more concretely if it is psychologically closer (Trope and Liberman, 2010). For example, by presenting detailed information to the viewers and links to protein supplements, the content creators make the idea of protein supplement consumption easy to explore. The information reduces the cognitive effort required of the audience and, consequently, the psychological distance for the audience to access protein supplements. This allows the viewer to think of protein supplements more concretely, leading them to purchase the product.

Finally, we can link “Psychological Reactance Theory” (PRT) to the overarching theme of “Genuineness.” The essence of PRT is that human beings enjoy their freedom and autonomy (Brehm, 1966). When individuals sense a threat to this freedom and autonomy, they experience a psychological reactance. Reactance refers to negative cognition resulting from a perceived threat to freedom (Reynolds-Tylus, 2019). Therefore, persuasive messages should be designed to be low-pressure, not pushy. PRT says that the conspicuousness of the underlying motives of a message may deteriorate the message's persuasive power. The theme “Genuineness” (Figure 3) entails codes that make the video creator appear more like a normal human being and less like a salesperson. By increasing relatability and creating trust in the video creator, it is possible to reduce the audience's reactance and encourage them to execute the action desired by the persuader.

One main difference we noticed when reviewing current research efforts (see the systematic literature review by Vrontis et al., 2021) was that the categories “Professional Expertise” (Step 1) and “Scientific Information” (overarching theme) contribute to persuasiveness in the context of YouTube and protein supplements. This might not seem surprising as TV celebrities like Dr Oz use similar strategies when advertising products to the public (Korownyk et al., 2014). The two categories are linked to the theme of “Genuiness” and “Quality”, thus, increasing any of these components increases the perceived genuineness of the SMI and advertised product. Interestingly, these factors seem to play less of a role in the context of fashion-related SMIs (Ladhari et al., 2020; Wiedmann and von Mettenheim, 2020).

### 5.2. Practical implications

We believe that our proposed three-step model can contribute to designing persuasive technology by offering a source of guidance and inspiration. Our model details three steps needed to persuade viewers on social media to click on a video, watch it and consequently act on the recommended advice. Overall, our model suggests that this persuasive process should not be seen as a single (e.g., Fogg, 2009) but rather a step-wise approach during which different influential features are at play. Our model could be used in digital marketing for designing persuasive advertisements but could also contribute to research efforts conducted in human-computer interaction.

For example, the development of virtual and artificial intelligence (AI) based influencers has attracted increased attention in research and commercial areas. This kind of disruptive technology requires a better understanding and customer engagement when used as a marketing strategy. Application areas in the context of social media marketing vary and range from identifying optimal promotion of products to providing customized support (Campbell et al., 2020).

Research has started to explore the use of AI to rank multimedia influencers based on audience and content representation to match them with marketeers (Farseev et al., 2018, 2021). In addition, the effect of AI and virtual SMIs on consumers has been investigated in different studies (Arsenyan and Mirowska, 2021; Block and Lovegrove, 2021; Sands et al., 2022). Arguably, such virtual influencers can fulfill a user's social needs just as good as any human could do (Arsenyan and Mirowska, 2021). However, findings indicate that (fashion) AI influencers are often perceived as less trustworthy (Sands et al., 2022).

The visual appearance of the virtual influencer seems to play a role in this context. For example, Arsenyan and Mirowska (2021) used computer-generated images (CGI) as part of a human-like virtual influencer in their study. Results indicate that such a human-like appearance can evoke a perception of “creepiness” in the human viewers. The authors speculate that creators of such applications should consider including aspects that make them appear more human and avoid a “picture-perfect” impression, which was perceived to affect the viewer's perception of the influencer as authentic. The authors suggest adding negative emotions to make them pass as humans. We argue that our model could provide further inspiration and guidance. For example, presenting the virtual SMI and product to be advertised in a slightly messy room could help them appear more relatable than being shown in a spotless living room with white couches. This could increase the perception of being relatable, potentially contributing to the genuineness of the advertisement, making it look less staged and more realistic.

The development of virtual influencers in an everyday context has become an increasingly popular topic. Despite being virtual, such SMIs can attract millions of followers (see Virtualhumans.org for an overview of different virtual influencers). Instagram alone has 35 verified virtual SMIs, including SMIs with a human-like appearance, such as “Lu of Magazine Luiza” (5.9 million followers), the brand representative for the Brazilian retail conglomerate Magazine Luiza. However, there are also several SMI with little resemblance to humans, including cartoon-style characters such as “Nobody sausage” (2.7 million followers) (Travers, 2022). These SMIs are, in some cases, official spokespersons of companies or have been sponsored by brands to endorse products on social media. For example, Miquela Sousa AKA “Lil Miquela” is a virtual CGI-generated influencer on Instagram who regularly endorses brands such as Prada (Sims, 2018; Yotka, 2018; Klein, 2020).

Some commercial applications have started to use AI SMIs as part of marketing approaches. For example, “PUMA Southeast Asia” partnered up in 2020 with “UM Studios x Ensemble Worldwide” to create a virtual AI-generated influencer named “Maya” to promote its “Future Rider” sneakers (Soulmachine, 2019). A year before, “YUMI” was developed by Soul Machines and the skincare brand SK-II as the world's first autonomously animated digital influencer providing beauty advice to the viewers and helping them understand their skin better. The creators indicate that to create a realistic experience, “hyper-real” images need to be combined with “hyper-real” conversations, responses and expressions (Soulmachine, 2019). Our model offers insights into additional persuasive features (e.g., humor as part of step two “Staying on the message”) that influence the experience and can be used as persuasive features. The use of our model could facilitate the process of conceptualizing and implementing future virtual and AI-based SMIs.

Combining our model with recent empirical research findings could contribute to the development process and provide concrete examples of successful ways to increase the persuasive influence of SMIs on YouTube. For example, results relating to the linguistic style used by human influencers could provide further guidance on how step three of our model named “concretization” can be effectively designed for (Munaro et al., 2021). Or how to enhance the attractiveness of YouTube video thumbnails that shows a synopsis of the video along with the title (Shimono et al., 2020), or automatically disclosing advertised content to viewers (Swart et al., 2020) to contribute to the perception of genuineness. As SMIs commonly endorse products to monetize their fame, are they required by law to disclose these relationships (FederalTradeComission, 2020). However, it seems that just a small percentage do so in practice (Mathur et al., 2018) and addressing such aspects could potentially help AI influencers mitigate aspects of appearing untrustworthy.

## 6. Limitations and future work

As with any research, there are some limitations to our findings. First, because this research is based on the interpretive paradigm, any generalization of this model must be carried out with extreme caution. As stressed before, the interpretivism philosophy holds that context is the key to understanding a phenomenon. Thus, whenever this model is used, the context of social media and digital platforms should be kept in mind. Translation to any other context, such as offline advertisement, should be accompanied by further research.

The second limitation is the subjective epistemology adopted during data analysis. Although we exercised a high degree of procedural precision, the results of this research are based on our interpretations which were inevitably influenced by our social experiences and knowledge. Furthermore, even though our model could contribute to the development of persuasive design, it should be noted that our model requires empirical testing to confirm its theoretical components. Furthermore, it is imperative to note that persuasiveness is subjective, which means that the persuasion's effectiveness will also depend on the audience receiving the message. It may, thus, be interesting to explore the extent to which this model applies to different demographics. And if there is a difference in the model's effectiveness due to demographics, it would be worth exploring the reasons for this difference.

Third, we selected search terms based on news reports and exploratory conversations with people who use YouTube to inform themselves about protein supplements. However, different search terms and consequent videos could reveal additional insights into persuasive features. We selected videos with a high view count. Nonetheless, including videos which have been recently published and have therefore a low view count could provide further insights. In addition, in the selection process, we did not consider how long ago the videos were published, which arguably has an influence on the number of views. Furthermore, it has been indicated that the day (weekday vs. weekend) and time of the video post (business hour vs. non-business hour) can influence a video's popularity (Munaro et al., 2021). We did not consider this aspect in our study.

Fourth, interviewing content creators (e.g., SMI), companies who advertise through YouTube and end consumers about the persuasive strategies displayed in the vlogs could provide further insights. Currently, this introspective view is missing from our model.

We presented our model to subject experts. Based on five open-ended interviews, we offer three suggestions for future work. First, the experts mentioned that examining factors that decrease persuasion on social media platforms might be interesting. Exploring such aspects could be combined into our model, which so far solely focuses on the factors that increase persuasiveness. Second, future research should also examine the relative importance of the categories included in the three steps, as well as the relative importance of each step in the overall effectiveness of a persuasive message. Finally, future studies could investigate if the proposed model translates to contexts outside of social media. For example, in political speeches or offline advertisements.

## 7. Conclusion

Social media advertisement often relies on influencers to promote services and products. This area has become a million-dollar industry using different persuasive strategies to make viewers follow their purchase advice. While research has focused on the area of beauty and fashion SMI, little is known about the techniques used to advertise products with associated health benefits. We proposed a three-step persuasion model for YouTube videos through an interpretive grounded theory methodology. While there are considerable amounts of persuasion theories that are in agreement with parts of the model, there is no single theory that combines all the factors we discovered into one model. Our model describes persuasion in this context as based on a multi-factorial process to make the viewer click on a video, watch it and act on the advice provided. Future empirical studies are needed to validate the theoretical components of our model.

## Data availability statement

The original contributions presented in the study are included in the article/supplementary material. Further inquiries can be directed to the corresponding author.

## Ethics statement

The studies involving human participants were reviewed and approved by University of Twente. The patients/participants provided their written informed consent to participate in this study.

## Author contributions

JT conducted the data collection, analysis, and wrote the first draft of the manuscript. RV and ML reviewed and revised the initial draft. Initial codes and themes were discussed among the three authors and refined. All authors have approved the final version of the manuscript.

## Funding

This research was funded by the Pride and Prejudice Project by the 4TU federation under Grant No. 4TU-UIT-346.

## Conflict of interest

The authors declare that the research was conducted in the absence of any commercial or financial relationships that could be construed as a potential conflict of interest.

## Publisher's note

All claims expressed in this article are solely those of the authors and do not necessarily represent those of their affiliated organizations, or those of the publisher, the editors and the reviewers. Any product that may be evaluated in this article, or claim that may be made by its manufacturer, is not guaranteed or endorsed by the publisher.
